# Prospective Study on Incidence, Risk Factors and Outcome of Recurrent *Clostridioides difficile* Infections

**DOI:** 10.3390/jcm10051127

**Published:** 2021-03-08

**Authors:** Guido Granata, Nicola Petrosillo, Lucia Adamoli, Michele Bartoletti, Alessandro Bartoloni, Gregorio Basile, Matteo Bassetti, Paolo Bonfanti, Raffaella Borromeo, Giancarlo Ceccarelli, Anna Maria De Luca, Stefano Di Bella, Sara Fossati, Erica Franceschini, Ivan Gentile, Daniele Roberto Giacobbe, Enrica Giacometti, Fabrizio Ingrassia, Filippo Lagi, Giambattista Lobreglio, Andrea Lombardi, Laura Isabella Lupo, Roberto Luzzati, Alberto Enrico Maraolo, Malgorzata Mikulska, Mario Umberto Mondelli, Alessandra Mularoni, Cristina Mussini, Alessandra Oliva, Alessandro Pandolfo, Carlotta Rogati, Filippo Fabio Trapani, Mario Venditti, Pierluigi Viale, Emanuela Caraffa, Maria Adriana Cataldo, on behalf of the ReCloDi (Recurrence of Clostridioides difficile Infection) Study Group

**Affiliations:** 1 Clinical and Research Department for Infectious Diseases, National Institute for Infectious Diseases L. Spallanzani IRCCS, 00149 Rome, Italy; guido.granata@inmi.it (G.G.); emanuela.caraffa@inmi.it (E.C.); adriana.cataldo@inmi.it (M.A.C.); 2 Infectious Diseases ISMETT IRCCS, 90127 Palermo, Italy; ladamoli@ismett.edu (L.A.); lucia.adamoli5@gmail.com (A.M.D.L.); amularoni@ismett.edu (A.M.); 3 Department of Medical and Surgical Sciences, “Alma Mater Studiorum”, IRCCS S. Orsola Teaching Hospital, University of Bologna, 40126 Bologna, Italy; bartolettimichele@yahoo.it (M.B.); f.f.trapani@gmail.com (F.F.T.); pierluigi.viale@unibo.it (P.V.); 4 Department of Experimental and Clinical Medicine, University of Florence, 50121 Florence, Italy; alessandro.bartoloni@unifi.it (A.B.); sciglianobasile@hotmail.it (G.B.); filippo.lagi@gmail.com (F.L.); 5 Department of Health Sciences (DISSAL), University of Genoa, 16126 Genoa, Italy; matteo.bassetti@unige.it (M.B.); daniele.roberto.giacobbe@gmail.com (D.R.G.); m.mikulska@unige.it (M.M.); 6 Infectious Diseases Unit, San Martino Polyclinic Hospital—IRCCS, 16132 Genoa, Italy; 7 Infectious Diseases Clinic, Department of Medicine University of Udine and Azienda Sanitaria Universitaria Integrata di Udine, 33100 Udine, Italy; enrica.giacometti@gmail.com; 8 Department of Infectious Diseases, San Gerardo Hospital, Monza—University of Milano-Bicocca, 20126 Milan, Italy; bonfanti.paolo@gmail.com; 9 Hospital of Crema, 26013 Crema, Italy; raffaellaborromeo@gmail.com (R.B.); fabrizio.ingrassia@asst-crema.it (F.I.); 10 Department of Public Health and Infectious Diseases, Sapienza University of Rome, Policlinico Umberto I, 00185 Rome, Italy; giancarlo.ceccarelli@uniroma1.it (G.C.); alessandra.oliva@uniroma1.it (A.O.); mario.venditti@uniroma1.it (M.V.); 11 Infectious Diseases Department, Azienda Sanitaria Universitaria Integrata di Trieste, 34128 Trieste, Italy; stefano932@gmail.com (S.D.B.); fossatisara@gmail.com (S.F.); roberto.luzzati@asuits.sanita.fvg.it (R.L.); 12 Clinic of Infectious Diseases, University of Modena and Reggio Emilia, 41121 Modena, Italy; ericafranceschini0901@gmail.com (E.F.); cristina.mussini@unimore.it (C.M.); carlotta.rogati@gmail.com (C.R.); 13 Section of Infectious Diseases, Department of Clinical Medicine and Surgery, University of Naples “Federico II”, 80138 Naples, Italy; ivan.gentile@unina.it (I.G.); albertomaraolo@mail.com (A.E.M.); 14 Vito Fazzi Hospital, 73100 Lecce, Italy; patologiaclinica.polecce@ausl.le.it (G.L.); lauraisalupo@gmail.com (L.I.L.); 15 Division of Infectious Diseases and Immunology, Fondazione IRCCS Policlinico San Matteo, 27100 Pavia, Italy; andrea.lombardi02@universitadipavia.it (A.L.); M.Mondelli@smatteo.pv.it (M.U.M.); 16 Infectious Diseases, ASST Lecco Hospital, 23900 Lecco, Italy; a.pandolfo@asst-lecco.it

**Keywords:** *Clostridioides difficile*, recurrence, risk factors, outcome, incidence

## Abstract

Background: Limited and wide-ranging data are available on the recurrent *Clostridioides difficile* infection (rCDI) incidence rate. Methods: We performed a cohort study with the aim to assess the incidence of and risk factors for rCDI. Adult patients with a first CDI, hospitalized in 15 Italian hospitals, were prospectively included and followed-up for 30 d after the end of antimicrobial treatment for their first CDI. A case–control study was performed to identify risk factors associated with 30-day onset rCDI. Results: Three hundred nine patients with a first CDI were included in the study; 32% of the CDI episodes (99/309) were severe/complicated; complete follow-up was available for 288 patients (19 died during the first CDI episode, and 2 were lost during follow-up). At the end of the study, the crude all-cause mortality rate was 10.7% (33 deaths/309 patients). Two hundred seventy-one patients completed the follow-up; rCDI occurred in 21% of patients (56/271) with an incidence rate of 72/10,000 patient-days. Logistic regression analysis identified exposure to cephalosporin as an independent risk factor associated with rCDI (RR: 1.7; 95% CI: 1.1–2.7, *p* = 0.03). Conclusion: Our study confirms the relevance of rCDI in terms of morbidity and mortality and provides a reliable estimation of its incidence.

## 1. Introduction

The Gram-positive anaerobic bacterium *Clostridioides difficile* (CD) is a leading cause of nosocomial diarrhea worldwide, resulting in significant morbidity, mortality and prolonged hospital stay [1,2,3]. Recurrence of *Clostridioides difficile* infection (rCDI) is associated with a higher risk of death and higher hospitalization costs [1,2,4,5,6]; subsequent rCDI episodes represent a real “spiral of disease”, and studies assessing the quality of life of patients with rCDI show that these patients live in constant concern of developing subsequent rCDI [7].

Clinical studies show wide-ranging rCDI rates after the primary CDI of 12% to 40% [8,9,10]. There is also an increased risk following any further recurrence of up to 64% [8,9,10,11,12]. However, scanty data are available on rCDI rates in Italy.

New, innovative treatment approaches, either antimicrobial or non-antimicrobial (e.g., monoclonal anti-toxin antibodies, microbiota transplantation, therapies with living bacteria and vaccines for CD), are in development. In the near future, these new therapies will represent effective alternatives in fighting and preventing rCDI. Therefore, efforts are needed to collect information on the burden of recurrence in wide populations in different countries and settings.

We performed a prospective multicenter cohort study with the objectives of assessing the 30-day onset rCDI rate in Italy, describing rCDI characteristics and outcomes and assessing the risk factors associated with rCDI.

## 2. Materials and Methods

### 2.1. Study Site, Design, Population and Variables

**Figure 1:**
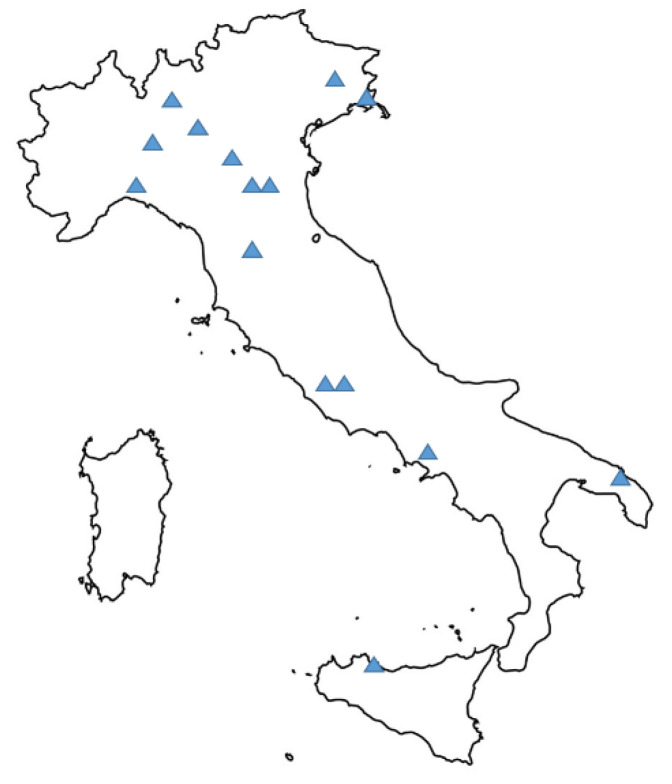
Geographical distribution of participating centers. A detailed list of the 15 participating centers is available as Supplementary Material (Table S1).

A prospective cohort study was performed in 15 Italian hospitals, including academic or tertiary referral hospitals. A complete list of participating centers is available as supplementary material (Supplementary Table S1), and their geographical distribution is shown in Figure 1. 

**Figure 2:**
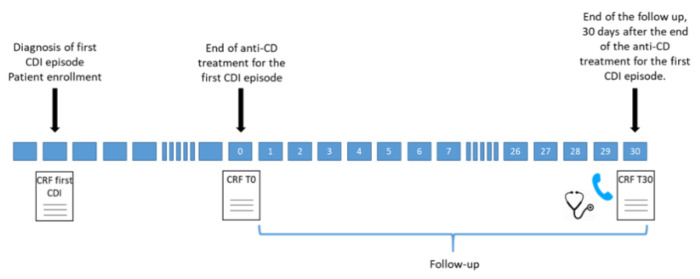
Details of the study follow-up. CDI: *Clostridioides difficile* infection. CRF: case report form.

All adult patients (age > 18 y) admitted to the participating centers from January 2018 to March 2020 with a diagnosis of a first CDI episode were included in the study [13]. All included patients were followed-up for 30 d after the end of antimicrobial treatment for their first CDI episode (detailed in Figure 2). 

For each enrolled patient, the following data were prospectively collected at study inclusion: age and gender; date of hospital admission; date of diarrhea onset and CDI diagnosis; hospitalization in the 3 months before the CDI diagnosis; treatment with antibiotics, antiacids, statins and steroids in the 3 months prior to CDI diagnosis; Charlson Comorbidity Index (CCI) at baseline and presence of co-morbidities. 

The following laboratory findings were registered at the time of CDI diagnosis: serum creatinine, serum albumin levels, white blood cell count and peripheral neutrophil count.

Data were also collected on the severity, treatment and outcome of the first CDI episode. 

During the 30-day follow-up, trained healthcare personnel assessed patients for the occurrence of rCDI; data on antibiotic exposure were also collected. In the case of hospital discharge before the end of follow-up, patients were contacted by phone call. Laboratory findings and clinical data of patients reporting rCDI diagnosis after hospital discharge were evaluated by the study investigators at the participating center, who decided whether or not to confirm the rCDI diagnosis according to the criteria reported below.

When a diagnosis of rCDI was confirmed during the 30-day follow-up period, data were collected on the severity and treatment of the rCDI episode. Available laboratory findings were also collected, including serum creatinine levels, serum albumin levels, white blood cell count and peripheral neutrophil count, at the time of onset of rCDI. Finally, mortality at the end of follow-up was recorded. 

To assess potential risk factors associated with rCDI, patients with rCDI were compared with patients who completed the follow-up and who did not present a recurrence of CDI in the 30-day period after the end of primary anti-CDI treatment. 

### 2.2. Definitions of CDI, Severe CDI and rCDI

CDI was diagnosed by considering both microbiological results and clinical information: (1) the presence of diarrhea or evidence of megacolon or severe ileus and (2) a positive laboratory diagnostic test result (e.g., toxin enzyme immunoassay (EIA) or nucleic acid amplification test (NAAT)).

Severe CDI was defined as an episode of CDI with the presence of at least one of the following criteria: fever (>38.5 °C), chills, hemodynamic instability, signs of ileus or peritonitis, leukocytosis (leukocytes > 15,000 cells/µL), increase in creatininemia > 1.5 times the value before infection onset, increase in serum lactates, evidence of pseudo-membranous colitis and radiological incidence of ileus or ascites [13].

In our study, CDI recurrence was considered when CDI re-occurred within 30 d after the end of treatment for the first CDI episode, provided symptoms from the previous episode resolved after completion of initial treatment.

### 2.3. Statistical Analysis

Quantitative variables were tested for normal distribution and compared by means of a two-tailed test. Differences in groups were assessed using a χ^2^ test and Fisher’s exact test. Precision of the risk ratio (RR) was determined by calculating a 95% confidence interval (95% CI). A p-value of less than 0.05 was considered statistically significant. Variables from univariate analyses were considered for inclusion in multivariate logistic regression analysis if p-values were less than 0.05. Backward stepwise logistic regression was performed, and the model that was considered biologically plausible and had the lowest −2 log–likelihood ratio was chosen as the final model. 

Statistical analysis was performed using the software program Inter-cooled Stata (Stata Statistical Software, version 15).

### 2.4. Ethical Considerations

The study was first approved by the Ethics Committee of the coordinating center (National Institute for Infectious Diseases “L. Spallanzani”, IRCCS, Rome; Ethics Committee registry number 543 23/01/2018) and, subsequently, by the Ethics Committees of the other 14 participating centers. Informed consent was obtained from each enrolled patient. 

## 3. Results

**Figure 3:**
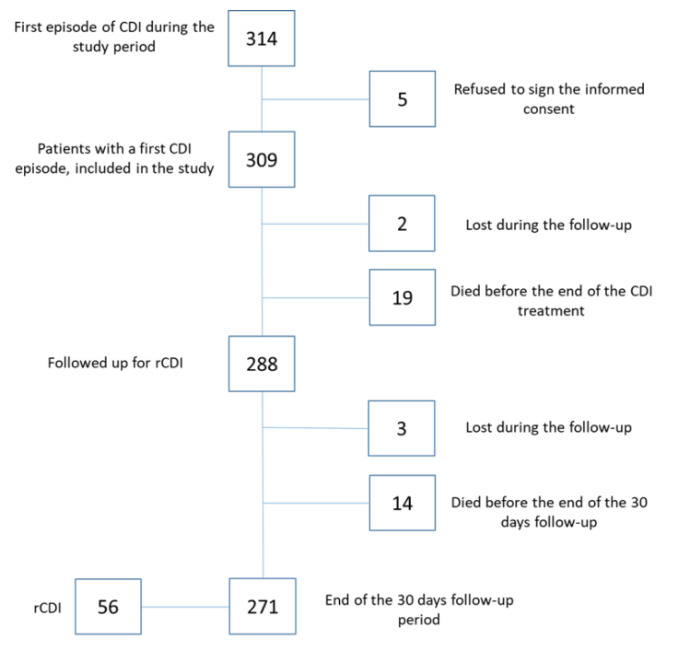
Follow-up of patients included in the study. CDI*: Clostridioides difficile* infection; rCDI: recurrent *Clostridioides difficile* infection.

Over the study period, 314 adult patients with a first CDI episode were enrolled; 309 patients gave informed consent and were included in the study. Two hundred eighty-eight patients were assessed at the end of antimicrobial treatment for their first CDI episode, and 271 patients completed the 30-day follow-up for a total of 7795 patient-days (Figure 3). 

### 3.1. Patients with a First CDI Episode

Table 1 shows the characteristics of 309 patients with a first CDI episode. Thirty-two percent of them (99/309) were severe/complicated.

Regarding patient outcomes, at the end of antimicrobial treatment for the first CDI episode, 153/309 (49.5%) patients were discharged home, 102/309 (33%) were still in the hospital and 35/309 (11.3%) were transferred to a long-term health-care facility, while 19/309 (6.1%) patients died during the CDI episode (Table 1).

Characteristics of the 288 CDI patients at the end of the anti-CDI treatment are shown in Table 2. For the first CDI episode, the most common anti-CDI antimicrobial was vancomycin (202/288, 70.1% of cases), followed by metronidazole (36/288, 12.5%) and a combination of vancomycin and metronidazole (33/288, 11.5%) (Table 2). At the end of antimicrobial treatment for the first CDI episode, 3/288 (1%) and 4/288 (1.4%) CDI patients needed surgery for complicated CDI and intensive care admission, respectively (Table 2). 

### 3.2. Patients with rCDI

Two hundred seventy-one patients completed the 30-day follow-up. During this period, a rCDI episode occurred in 56 of them (21%), with an incidence rate of 71.8 episodes per 10,000 patient-days. 

Table 3 shows the characteristics of the 56 rCDI patients. 

Their mean age was 70 y (range 29–85); 89% of them had one or more co-morbidities (50/56). In 22 of them (39%), their first CDI episode was severe or complicated. 

During the follow-up period, 22/56 rCDI patients (39.2%) received antibiotics. Only in 14 (63.6%) of these was it possible to assess indication for antibiotic treatment, including pneumonia, abdominal infection, tuberculosis, osteomyelitis and urinary tract infection in 5 (22.7%), 4 (18.2%), 3 (13.6%), 1 (4.5%) and 1 (4.5%) of the patients, respectively.

Thirteen (23.2%) rCDI episodes were defined as severe/complicated. Treatment comprised vancomycin, fidaxomicin, a combination of vancomycin plus metronidazole and metronidazole in 34 (60.7%), 10 (17.8%), 7 (12.5%) and 2 (3.5%) cases, respectively. Seven out of 56 (12.5%) rCDI cases also received albumin during anti-rCDI treatment (Table 3).

Regarding the rCDI outcome, 50 out of 56 (89.3%) rCDI cases recovered. Six out of 56 (10.7%) patients died.

### 3.3. Risk Factors for rCDI

The results of the univariate analysis are shown in Table 4. Logistic regression analysis identified exposure to cephalosporin during the 30-day follow-up and/or 3 months prior to the first CDI episode as the sole independent risk factor associated with the occurrence of rCDI (RR: 1.7; 95% CI: 1.1–2.7, *p* = 0.03). 

Finally, at the end of the follow-up period, overall crude mortality rate (either in-hospital or post-discharge) was 10.7% (33/309).

## 4. Discussion

Our findings confirm that rCDI represents a relevant problem in terms of morbidity, mortality and impact on public health. Indeed, in our cohort, the incidence rate of rCDI was 72 cases per 10,000 patient-days; 21% (56/271) of patients with a first CDI episode developed rCDI.

There are limited data on rCDI incidence. Importantly, studies assessing rCDI incidence differ by type of cohorts included and, specially, by follow-up periods. Furthermore, most of the information on the rate of rCDI comes from studies aimed at evaluating the efficacy of anti-CDI therapies, rather than from studies specifically designed to determine it. As a consequence, rCDI incidence rates are wide-ranging [8,9,10], and the transferability of findings from studies assessing this rate is low. 

In our cohort, the inclusion of all hospitalized patients with a first CDI episode and the rigorous methodology for assessment of recurrences allowed a trustworthy estimation of the incidence rate. We believe that reporting the incidence rate per 10,000 patient-days gives a better definition of the extent of the problem than simply reporting the percentage of rCDI patients. We are not aware of published studies that provide this information, which would make it easier to compare incidences in different cohorts. 

One of the main limitations of our study pertains to the follow-up period. According to international guidelines, CDI recurrence is defined as a CDI episode that re-occurs within eight weeks after the onset of the previous CDI episode, provided symptoms from the previous episode resolve after completion of initial treatment. Our follow-up period was shorter than eight weeks after the onset of the first CDI episode; however, we preferred to consider a different time period for two main reasons: (1) the 30 days after the end of anti-CDI treatment is the period in which relapses occur most frequently [14,15]; (2) we ensured that the follow-up period was the same for all enrolled patients. In our cohort, the mean time from the end of anti-CD treatment for the first CDI episode to the new onset of diarrhea in rCDI patients was 18 ± 9 days. However, it is important to consider that we could have missed some cases if recurrence occurred more than 30 days after the end of CDI treatment.

Another limitation of the study is the mean age (70 y) of the patient population. Old age was stated as a risk factor for rCDI in many countries, and the relatively high mean age of this study population may have affected results. However, it should be considered that the study population reflects consecutive CDI cases enrolled in the 15 academic or tertiary referral Italian hospitals participating in the study. 

Regarding risk factors, in our study only previous exposure to cephalosporins was independently associated with the risk of rCDI. In the literature, there are several studies evaluating the role of antibiotic exposure; antibiotics most frequently recognized as predisposing factors for CDI include fluoroquinolones, clindamycin and broad-spectrum penicillins and cephalosporins [16]. Regrettably, studies have evaluated different periods of exposure to antibiotics; thus, this information is frequently biased. The protective effect of penicillins that we found in our univariate analysis is difficult to explain. The majority of these patients received penicillins in the three months prior to their first CDI episode, while the percentages of rCDI and not-rCDI patients receiving penicillins during the 30-day follow-up decreased to 7% and 10%, respectively. The univariate analyses limited to the 30-day follow-up found no significant differences between the two groups.

Our findings highlight the importance of antibiotic exposure, before the first CDI episode and after its resolution, in determining long-lasting effects on gut microbiota leading to recurrence of CDI. Physicians should sharpen their clinical judgment when choosing antibiotic therapy and strongly reinforce compliance with basic antimicrobial stewardship principles. 

Finally, our study gives additional information on the outcomes of patients with primary CDI and rCDI.

Our findings on mortality are coherent with other published data; according to a meta-analysis, patients with CDI have a risk of 30-day mortality ranging between 8% and 53% [10]. Regarding the mortality rate of rCDI patients, previous studies reported rates of 9.3% [17] and 7.8% at 30 days after recurrence [18].

In conclusion, our study adds further insight into the characteristics, incidence rate, risk factors and mortality rate of rCDI. Further studies should assess the risk of antibiotic exposure by analyzing the risk associated with each antibiotic class in-depth and defining the time of exposure which should be considered at-risk. Providing the incidence rate for 10,000 days of follow-up would allow comparable and reliable data on rCDI to be obtained. 

## Figures and Tables

**Table 1:** Baseline characteristics of the 309 patients with a first CDI included in the study. CCI: Charlson Comorbidity Index; SD: standard deviation; CDI: *Clostridioides difficile* infection; COPD: chronic obstructive pulmonary disease; LTCF: long-term care facility. SD: standard deviation.

Variables	CDI Patients (%)
Female gender	152 (46.2%)
Age (y)	70 (range: 18–95)
Hospital onset of CDI	168 (54.4%)
Comorbidities	286 (92.6%)
Cardiovascular disease	168 (54.3%)
Heart failure	55 (17.8%)
Diabetes	75 (24.2%)
Renal failure	57 (18.4%)
Dialysis	12 (3.98%)
Chronic liver failure	30 (9.1%)
Neurological disease	52 (16.8%)
Vasculitis	9 (2.9%)
COPD	69 (22.3%)
Solid cancer	42 (13.6%)
Hematologic cancer	23 (7.4%)
Transplant, immunodeficiency, immunosuppression	39 (12.6%)
Other concomitant infections	103 (33.3%)
Mean age-adjusted CCI	5.3 (range: 0–13)
Hospitalization in the previous 3 months	164 (53%)
Transferred to the hospital from a LTCF	34 (11%)
Antibiotic administration in the previous 3 months	220 (71.2%)
Antiacids administration in the previous 3 months	214 (69.2%)
Statins administration in the previous 3 months	77 (24.9%)
Steroids administration in the previous 3 months	85 (27.5%)
Mean baseline serum creatinine (mg/dL ± SD)	1.4 ± 1.4
Mean baseline serum albumin (g/dL ± SD)	2.9 ± 0.7
Antibiotic administration at CDI diagnosis	191 (61.8%)
CDI severity at diagnosis	
Mild CDI	210 (68.0%)
Severe/complicated CDI	99 (32.0%)
Mean laboratory findings at CDI diagnosis	
Total peripheral white blood cell count (10^3^ cells/µL ± SD)	12.03 ± 9.40
Peripheral neutrophil count (10^3^ cells/µL ± SD)	9.64 ± 7.85
Serum creatinine (mg/dL ± SD)	1.4 ± 1.4
Serum albumin (g/dL ± SD)	2.9 ± 0.7

**Table 2:** Characteristics of the 288 patients with a first CDI at the end of anti-CDI treatment for their first CDI episode and resolution of diarrhea. SD: standard deviation. PO: per os. IV: intravenous administration.

Variables	Number of CDI Patients (%)
Administered anti-CD antimicrobial treatment	
Vancomycin	202 (70.1%)
PO Metronidazole	36 (12.5%)
Vancomycin and IV metronidazole	33 (11.5%)
Vancomycin and IV metronidazole and fidaxomicin	5 (1.7%)
Vancomycin and fidaxomicin	7 (2.4%)
Vancomycin and tigecycline	2 (0.6%)
Fidaxomicin	1 (0.3%)
Vancomycin and IV metronidazole and tigecycline	2 (0.6%)
Albumin administration	35 (12.1%)
Surgery for complicated CDI	3 (1%)
Necessity of intensive care	4 (1.4%)
Mean length of hospital stay of the discharged patients (d)	23 (range: 1–167)

**Table 3:** Outcomes and characteristics of the 56 rCDI patients. SD: standard deviation. rCDI: recurrent *Clostridioides difficile* infection. PO: per os. IV: intravenous administration.

Variables	Number of rCDI Patients (%)
rCDI severity at diagnosis	
Mild	43 (76.8%)
Severe/complicated	13 (23.2%)
Mean time from the end of anti-CD treatment for the first CDI episode to the onset of rCDI diarrhea (days ± SD)	17.8 ± 8.6
Administered anti-CD antimicrobial treatment for rCDI	
Vancomycin	34 (60.7%)
PO Metronidazole	2 (3.5%)
Vancomycin and IV metronidazole	7 (12.5%)
Fidaxomicin	10 (17.8%)
Other	3 (5.3%)
Laboratory findings at rCDI diagnosis	
Total peripheral white blood cell count (10^3^ cells/µL ± SD)	11,886 ± 7912
Peripheral neutrophil count (10^3^ cells/µL ± SD)	8834 ± 6828
Serum creatinine (mg/dL ± SD)	1.5 ± 1.2
Serum albumin (g/dL ± SD)	3.3 ± 0.6
Albumin administration after rCDI diagnosis	7 (12.5%)
Surgery for complicated rCDI	1 (1.7%)
Necessity of intensive care	1 (1.7%)
Outcome of rCDI	
Recovered at home	50 (89.3%)
Deceased, rCDI related	6 (10.7%)

**Table 4:** Risk factors for the occurrence of rCDI. Univariate analysis. RR: risk ratio. CI: confidence interval. SD: standard deviation. rCDI: recurrent *Clostridioides difficile* infection. PO: per os. IV: intravenous administration.

Variables	rCDI Patients (N = 56) (%)	Not rCDI Patients (N = 215) (%)	RR (95% CI)	*p*
Mean age (y ± SD)	72 ± 15	67 ± 17	--	0.08
Female gender	28 (50%)	98 (45.6%)	1.2 (0.7–1)	0.6
Anti-CD treatment for the first CDI episode				
Vancomycin monotherapy	38 (67.8%)	153 (71.2%)	0.9 (0.5–1.5)	0.6
PO metronidazole monotherapy	9 (16.6%)	23 (10.7%)	1.4 (0.8–2.6)	0.3
Fidaxomicin (alone, in combination)	0	12 (5.6%)	--	0.07
Vancomycin plus IV metronidazole	6 (10.7%)	30 (13.9%)	0.8 (0.4–1.7)	0.5
Bezlotoxumab	1 (1.8%)	1 (0.5%)	0.4 (0.1–1.7)	0.3
First CDI episode severe/complicated	22 (39.3%)	59 (27.4%)	1.5 (0.9–2.4)	0.08
Antibiotics administration during 30-day follow-up	22 (39.3%)	74 (34.4%)	1.2 (0.7–1.9)	0.5
Antibiotics administration during 30-day follow-up and/or 3 months prior to the first CDI episode	52 (92.8%)	198 (92.1%)	1.1 (0.4–2.7)	0.8
Quinolones	17 (30.3%)	45 (20.9%)	1.5 (0.9–2.4)	0.1
Penicillins	20 (35.7%)	112 (52.1%)	0.6 (0.4–1)	0.03
Carbapenems	10 (17.8%)	38 (17.7%)	1 (0.5–1.9)	1
Cephalosporins	30 (53.5%)	74 (34.4%)	1.9 (1.2–2.9)	0.009
Glycopeptides	7 (12.5%)	25 (11.6%)	1.1 (0.5–2.2)	0.9
Clindamycin	1 (1.8%)	3 (1.4%)	1.2 (0.2–6.7)	0.8

## Data Availability

The data presented in this study are available on request from the corresponding author. The data are not publicly available due to privacy restrictions.

## Supplementary Materials

The following are available online at https://www.mdpi.com/2077-0383/10/5/1127/s1, Table S1: List of participating centers.
