# Supplementary material for: Prospective Study on Incidence, Risk Factors and Outcome of Recurrent Clostridioides difficile Infections
Source: J Clin Med. 2021 Mar 8;10(5):1127. doi: 10.3390/jcm10051127 (PMC7962640; doi:10.3390/jcm10051127)
Supplement: Supplementary file 1 [file jcm-10-01127-s001.pdf]

Table S1. List of participating centres and number of CDI cases included in the study by each centre. CDI: *Clostridioides difficile* infection.

**Centers**	**Number of included CDI cases**	**Female gender N (%)**	**Mean age, years**
IRCCS Istituto Nazionale Malattie Infettive "L. Spallanzani"	71	32 (45)	60
AO Ospedale Maggiore Di Crema; Crema	12	6 (50)	82
AOU Careggi; Florence	19	9 (47)	72
AOU Federico II di Napoli; Naples	7	4 (57)	72
AOU Policlinico di Modena; Modena	32	23 (72)	72
Azienda Sanitaria Universitaria Integrata di Trieste; Trieste	21	12 (57)	75
Azienda Sanitaria Universitaria Integrata di Udine; Udine	5	2 (40)	75
Azienda Socio Sanitaria Territoriale di Lecco; Lecco	6	3 (50)	80
Sapienza University, Policlinico Umberto I; Rome	45	19 (42)	73
ISMETT IRCCS; Palermo	29	11 (38)	59
Ospedale Policlinico San Martino – IRCCS; Genoa	10	5 (50)	78
Policlinico Sant'Orsola-Malpighi; Bologna	17	10 (59)	70
Ospedale Maggiore; Bologna	5	--	74
Vito Fazzi General Hospital; Lecce	10	3 (30)	74
IRCCS "San Matteo", University of Pavia; Pavia	20	10 (50)	69
